# Supplementary material for: Circumventing Drug Treatment? Intrinsic Lethal Effects of Polyethyleneimine (PEI)-Functionalized Nanoparticles on Glioblastoma Cells Cultured in Stem Cell Conditions
Source: Cancers (Basel). 2021 May 27;13(11):2631. doi: 10.3390/cancers13112631 (PMC8198814; doi:10.3390/cancers13112631)
Supplement: Supplementary file 1 [file cancers-13-02631-s001.zip › supplementary material/cancers-1180960-final-suppl-done.pdf]

cancers

MDPI

Supplementary Material: Circumventing Drug Treatment? Intrinsic Lethal Effects of Polyethyleneimine (PEI)-Functionalized Nanoparticles on Glioblastoma Cells Cultured in Stem Cell Conditions

Neeraj Prabhakar, Joni Merisaari, Vadim Le Joncour, Markus Peurla, Didem Şen Karaman, Eudald Casals, Pirjo Laakkonen, Jukka Westermarck and Jessica M. Rosenholm

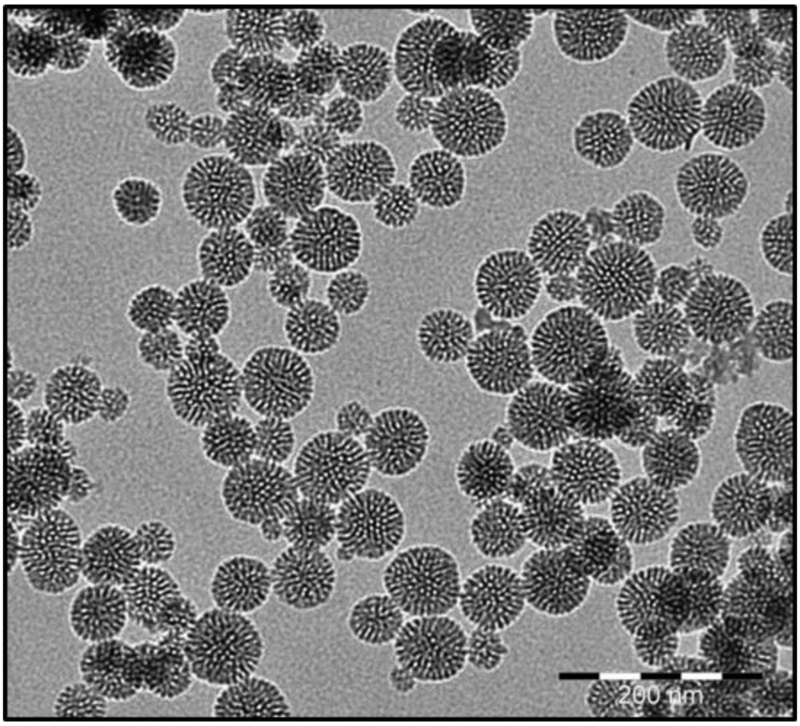

Figure S1. TEM micrograph of the spherical PEI-MSNs particles with an approximate size of 50 nm with mesoporous structure. Scale bar = 200 nm.

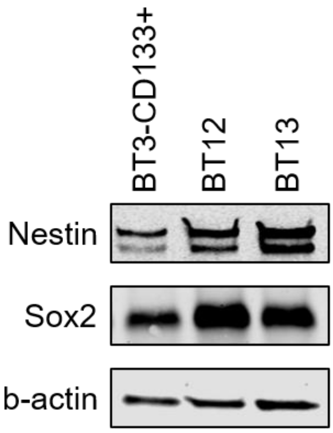

Figure S2. Validation of GSCs (BT-3-CD133^{+}, BT-12, and BT-13 (cultured in stem cell conditions) using Sox2 and nestin (stem cell markers). The original western blot figures are shown in File S1.

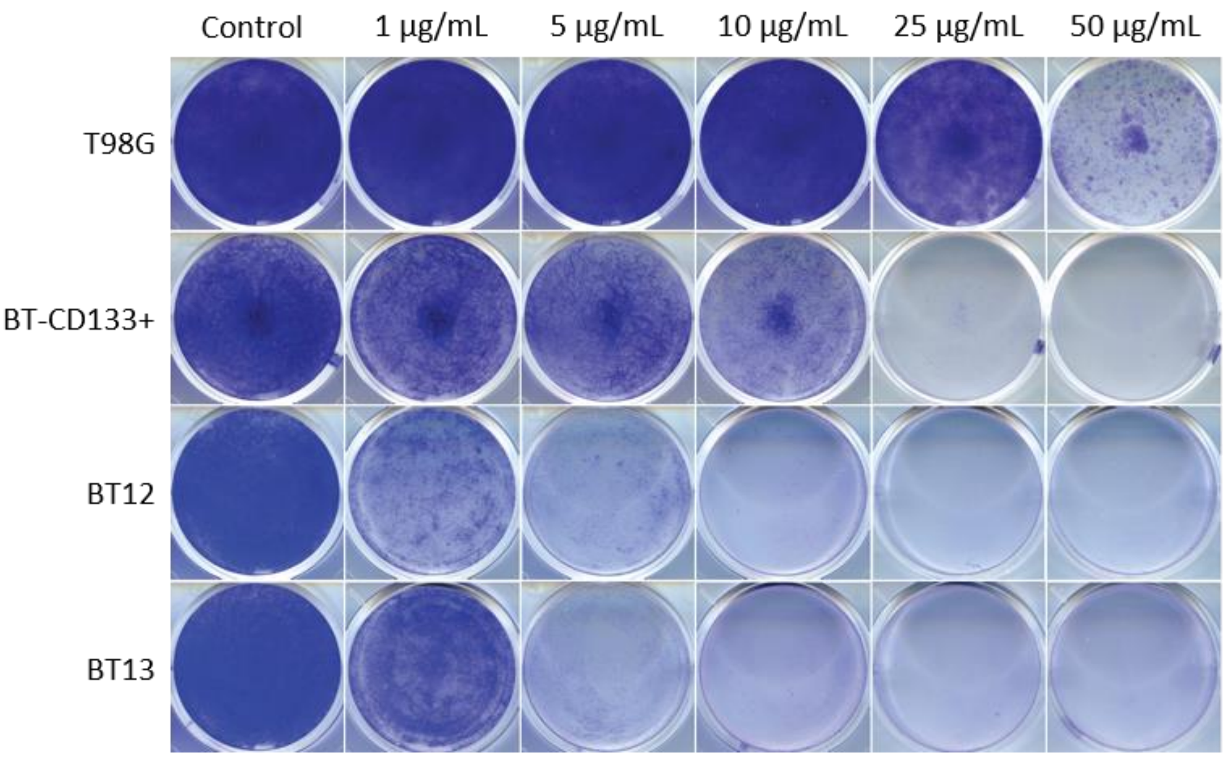

Figure S3. Selective cell death of patient-derived GSCs by PEI-MSNs. Colony growth assay analysis by using crystal violet staining of T98G (cultured in standard conditions) (GB cell line), BT-3-CD133^{+}, BT-12 and BT-13 (cultured in stem cell conditions) GSCs treated with 1-50 µg/mL of PEI-MSNs.

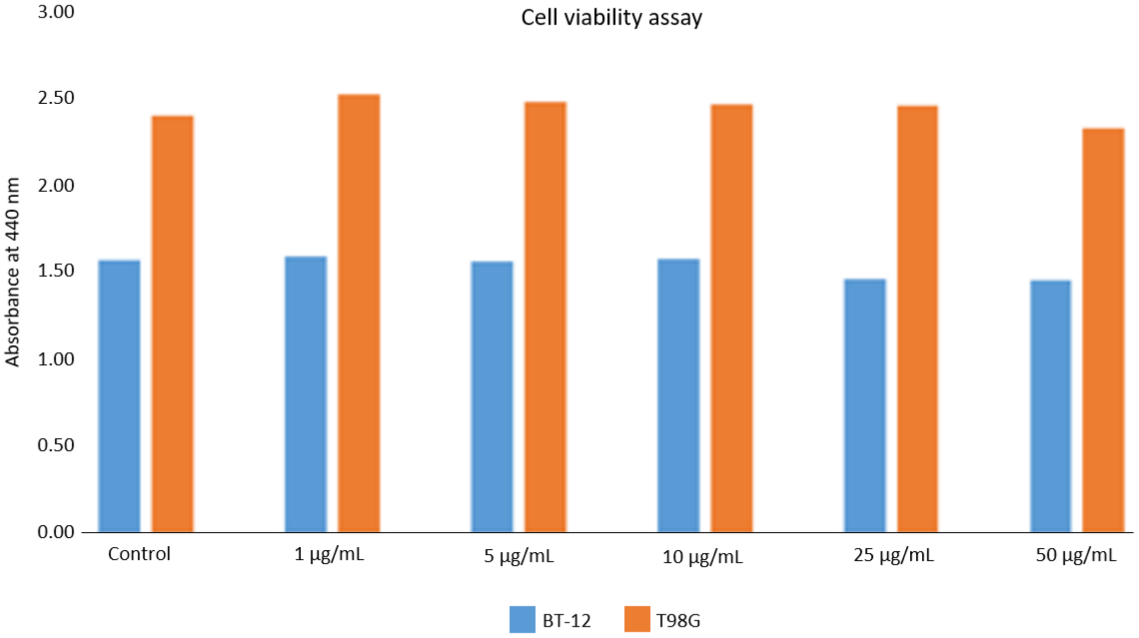

Figure S4. Viability of BT-12 GSCs (cultured in stem cell conditions) and T98G (cultured in standard conditions) (GB) cells treated with 1-50µg/mL of plain MSNs (without PEI). The cell viability was assessed by WST-1 cell proliferation assay.

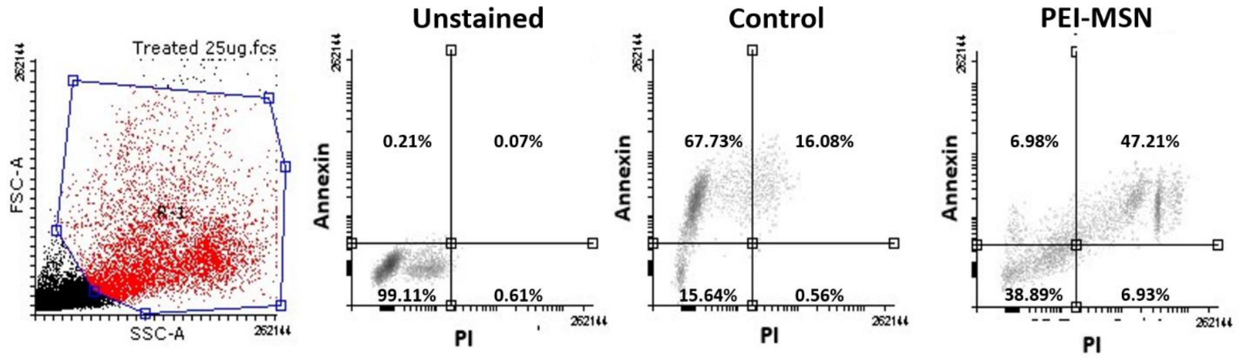

Unstained:

	Events	% of Vis
All events	12,209	100.00
Left Bottom	12,100	99.11
Right Bottom	75	0.61
Left Top	26	0.21
Right Top	8	0.07

Control:

	Events	% of Vis
All events	12,056	100.00
Left Bottom	1,885	15.64
Right Bottom	67	0.56
Left Top	8,165	67.73
Right Top	1,939	16.08

PEI-MSN:

	Events	% of Vis
All events	13,333	100.00
Left Bottom	5,185	38.89
Right Bottom	924	6.93
Left Top	930	6.98
Right Top	6,294	47.21

Figure S5. Cell death analysis by flow cytometry. Annexin V was used as a marker to detect apoptotic cells and Propidium iodide (PI) for cell viability. Consistent with cleaved PARP analysis (Figure 4A) the non-treated BT3-CD133^{+} (cultured in stem cell conditions) cells (Control) demonstrated apoptotic cell population. This population was rather decreased than increased in the PEI-MSN treated cells (Treated).

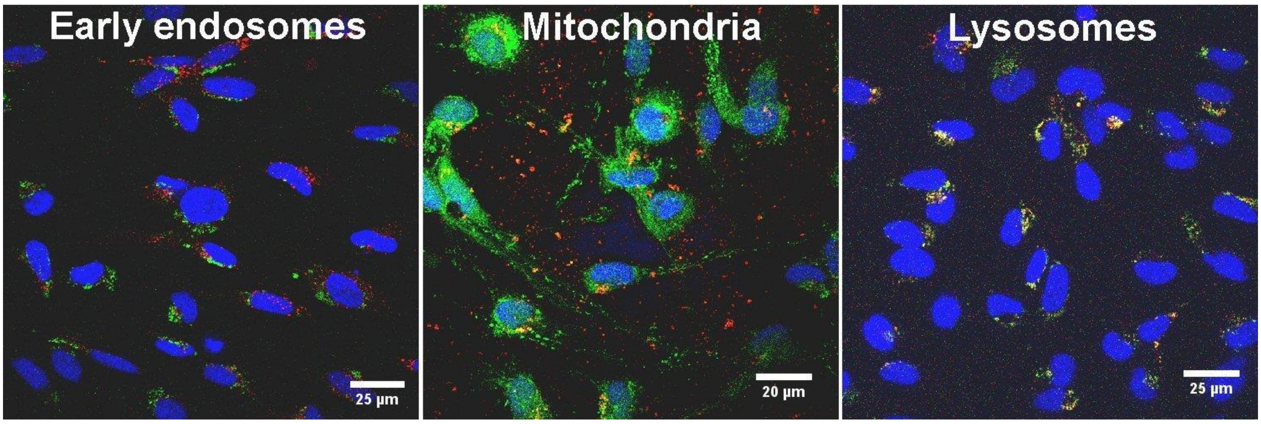

Figure S6. Localization of the PEI-MSNs in the treated BT-12 GSCs (cultured in stem cell conditions) by confocal microscopy. Intracellular localization of PEI-MSNs (red) was studied using markers of early endosomes (EEA-1), mitochondria (Mitotracker), and lysosomes (LAMP-1) (green color). The nuclei were visualized by using DAPI (blue). Co-localization is seen in yellow color.

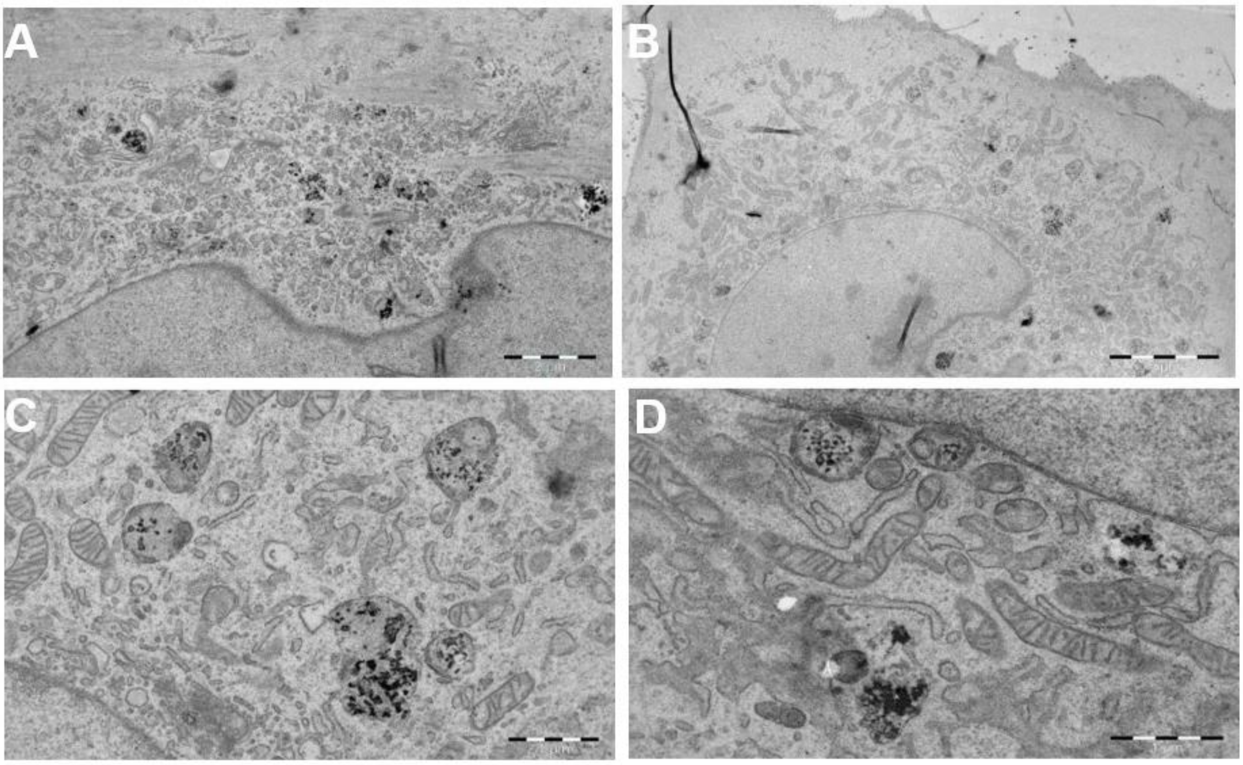

Figure S7. TEM images of PEI-MSNs treated MDA-MB-231 (cultured in standard conditions) after 72h. (**A**–**B**) PEI-MSNs remained confined to vesicular structures and no visible damage was observed to cellular ultrastructure. (**C**–**D**) TEM imaging suggests that PEI-MSNs treated MDA-MB-231 cells did not exhibit any visible cytotoxic effects to mitochondria, lysosomes in comparison to BT-12 cells (Stem cells).

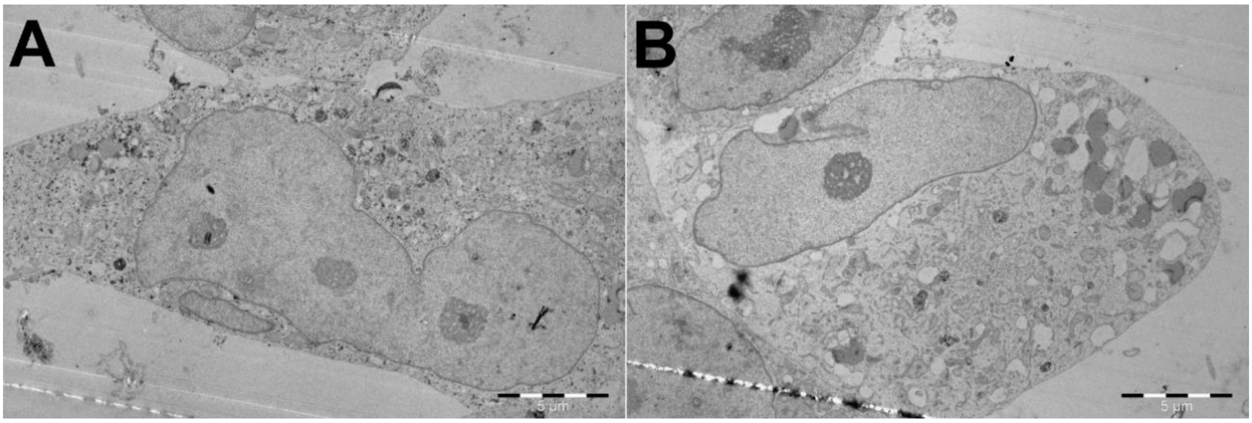

Figure S8. TEM images of PEI-MSNs treated BT-12 (cultured in stem cell conditions) GSCs after 72h. (**A**–**B**) No PEI-MSNs were detected within the nuclear space.

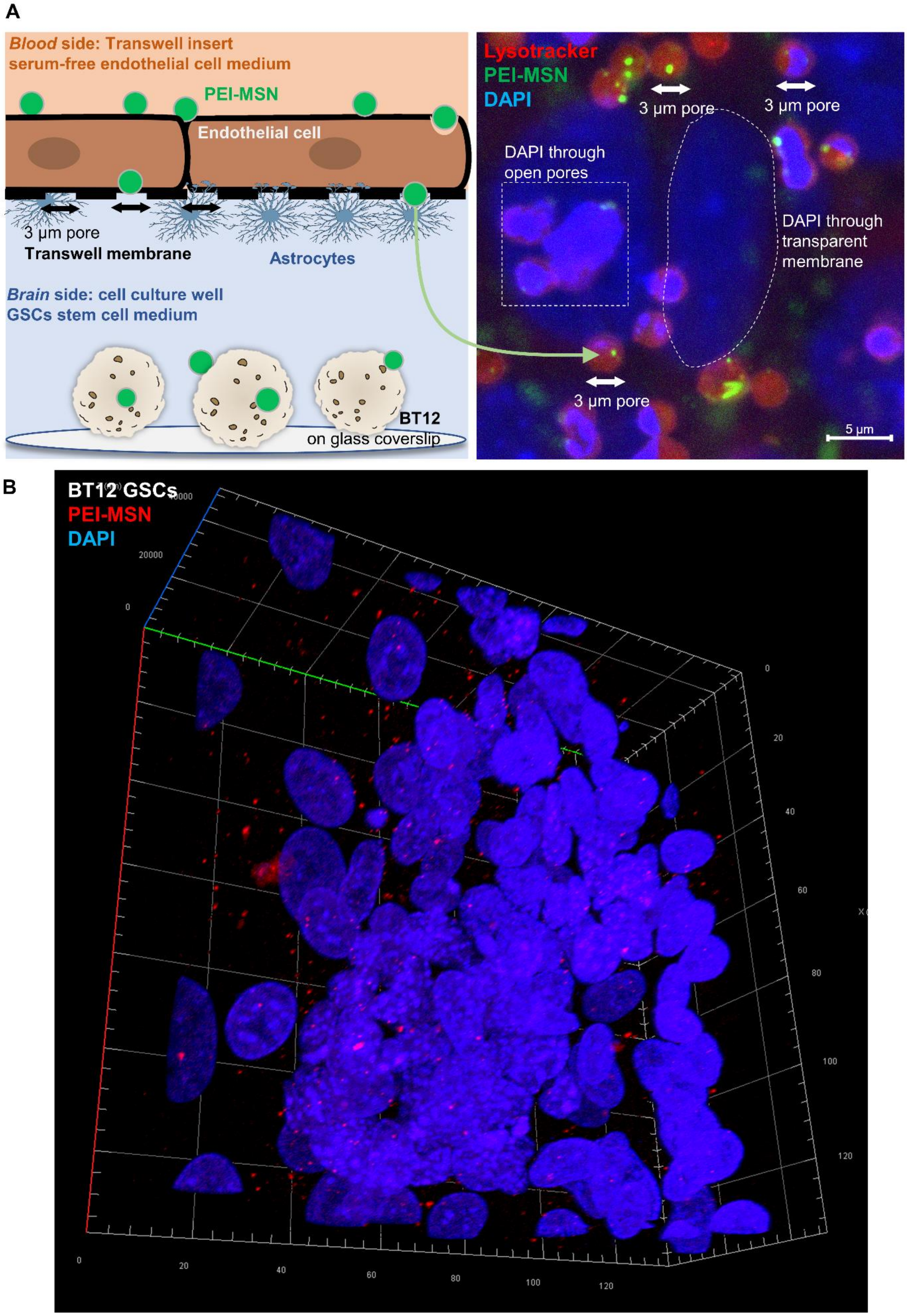

Figure S9. (**A**) Schematic representation of the BBTB assay (left) and sagittal confocal bottom view of the Transwell membrane as seen from the brain side. Endothelial cells (lysotracker, red) nuclei (DAPI, blue) from the blood side can be seen through the semi-tranparent membrane (circled) and occasionally across the 3 µm pores (dotted square). PEI-MSN (green)

transcytosis from the blood to the brain sides is observable through the pores. (**B**) 3D reconstruction from confocal Z-stacks of a BT-12 GSC isolated from the BBTB. PEI-MSNs (red) are distributed all around and penetrated inside the sphere.
