# Supplementary material for: Circumventing Drug Treatment? Intrinsic Lethal Effects of Polyethyleneimine (PEI)-Functionalized Nanoparticles on Glioblastoma Cells Cultured in Stem Cell Conditions
Source: Cancers (Basel). 2021 May 27;13(11):2631. doi: 10.3390/cancers13112631 (PMC8198814; doi:10.3390/cancers13112631)

Figure 4.

- Samples run in dublicates
- All data are same from the same membrane.
- Between each picture the membrane has been stripped and reprobed.

BT12

Control 24h 48h

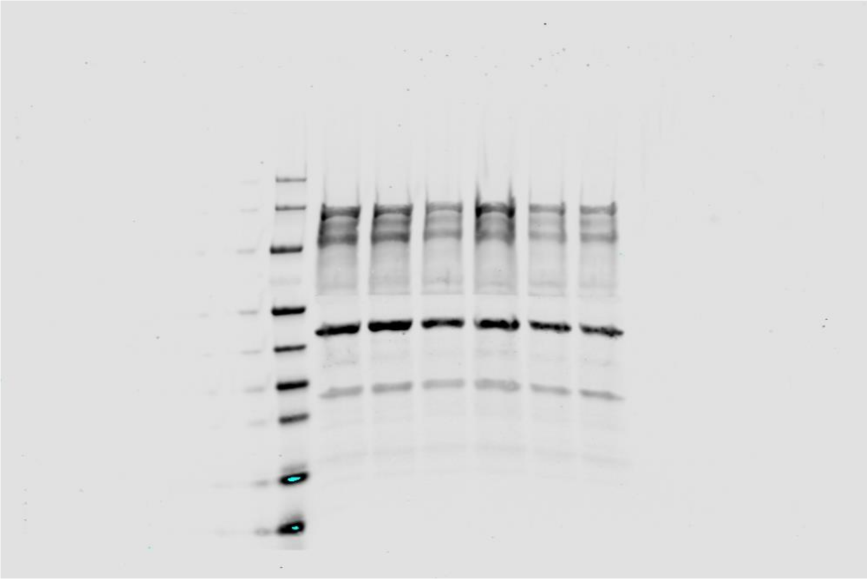

Full PARP-1 (116 kDA)

cPARP-1 (89kDa)

B-actin (42 kDa)

cPARP-1 (25kDa)

BT12

Control 24h 48h

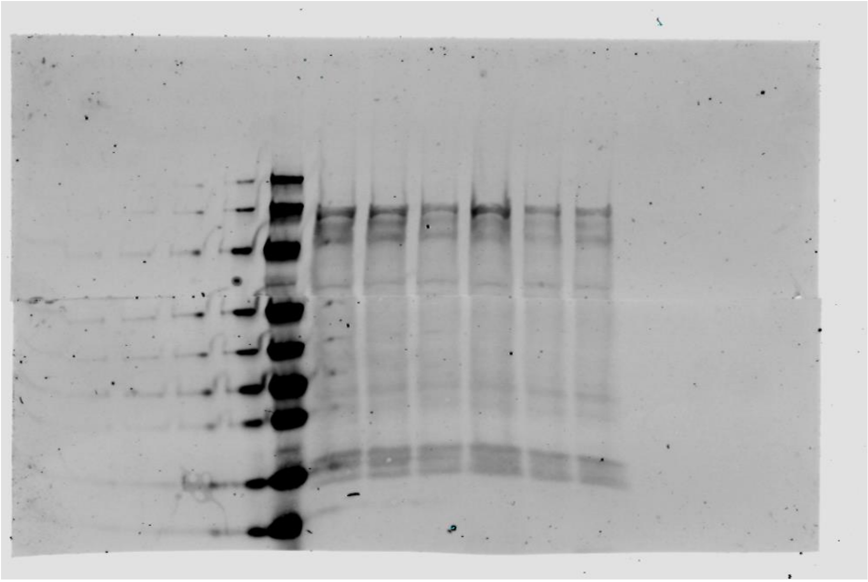

LC3B (15 kDa)

BT12

Control 24h 48h

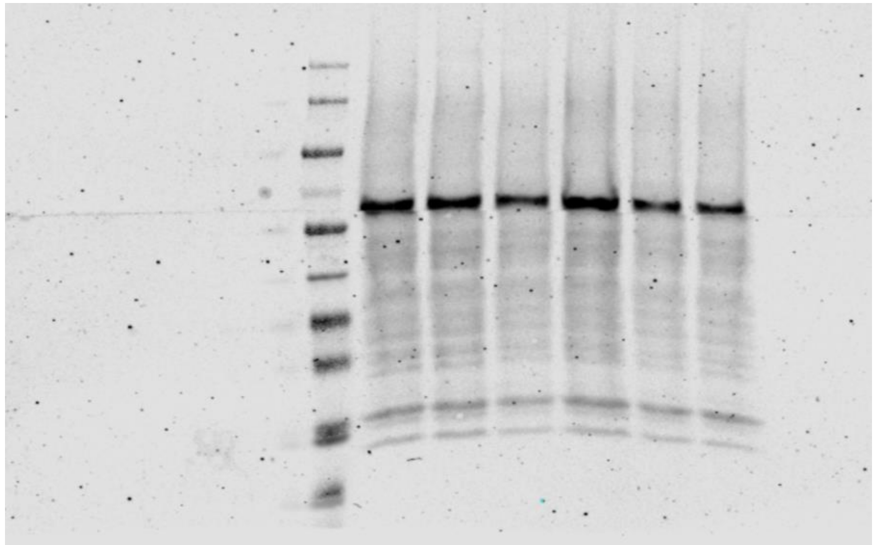

P62 (65 kDa)

Supplementary figure 2.

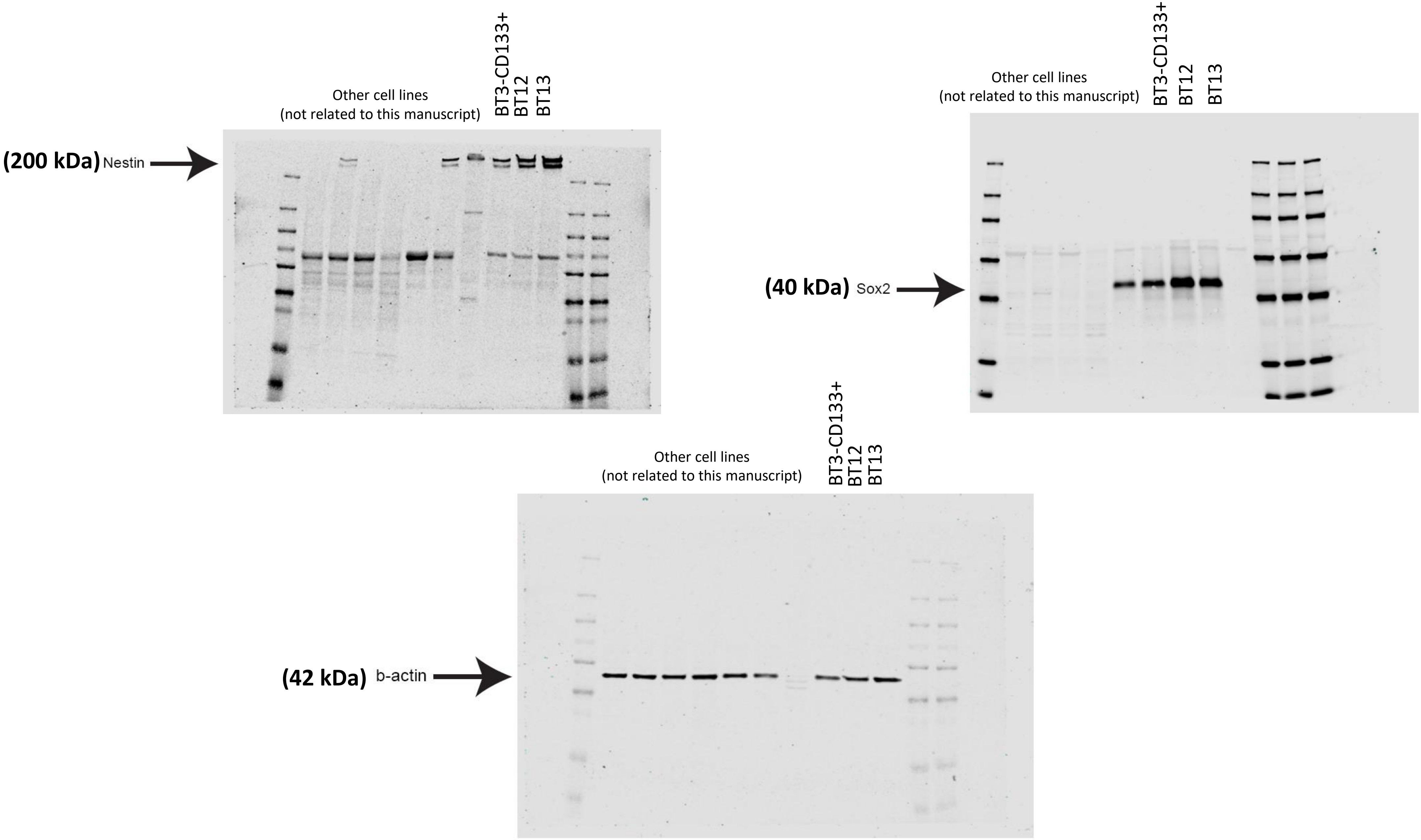

Supplement: Supplementary file 1 [file cancers-13-02631-s001.zip › supplementary material/File S1. Original western blot figures.pdf]
